# What parameters affect left ventricular diastolic flow propagation velocity? in vitro studies using color m-mode doppler echocardiography

**DOI:** 10.1186/1476-7120-3-24

**Published:** 2005-09-01

**Authors:** Toshihiro Ogawa, Lawrence N Scotten, David K Walker, Ajit P Yoganathan, Renee L Bess, Cheryl K Nordstrom, Julius M Gardin

**Affiliations:** 1 St. John Hospital & Medical Center, Detroit, MI, USA; 2 Vivitro Systems, Inc., Victoria, BC, Canada; 3 Georgia Institute of Technology, Atlanta, GA, USA

## Abstract

**Background:**

Insufficient data describe the relationship of hemodynamic parameters to left ventricular (LV) diastolic flow propagation velocity (Vp) measured using color M-mode Doppler echocardiography.

**Methods:**

An in vitro LV model used to simulate LV diastolic inflow with Vp measured under conditions of varying: 1) Stroke volume, 2) heart rate (HR), 3) LV volume, 4) LV compliance, and 5) transmitral flow (TMF) waveforms (Type 1: constant low diastasis flow and Type 2: no diastasis flow).

**Results:**

Univariate analysis revealed excellent correlations of Vp with stroke volume (r = 0.98), LV compliance (r = 0.94), and HR with Type 1 TMF (r = 0.97). However, with Type 2 TMF, HR was not associated with Vp. LV volume was not related to Vp under low compliance, but inversely related to Vp under high compliance conditions (r = -0.56).

**Conclusion:**

These in vitro findings may help elucidate the relationship of hemodynamic parameters to early diastolic LV filling.

## Background

The flow Vp of early diastolic LV inflow measured using color M-mode Doppler echocardiography (CMD) has been recognized as a useful measure of LV relaxation. [1-4] Vp by CMD has also been reported to correlate with the time constant of isovolumic relaxation (τ). [1,3,4] However, there are few published data describing the relationship of hemodynamic parameters to Vp. In the clinical and in vivo experimental situations, it is difficult to evaluate the influence of any single hemodynamic variable on Vp because changing one variable often is associated with changes in other variables, such as LV contraction and heart rate. Therefore, to better understand the influence of changes in various hemodynamic parameters on Vp, we performed in vitro CMD studies using a mechanical LV model which allowed us to produce isolated changes in the hemodynamic parameters.

## Methods

### Model

We used a customized commercially-available LV model (Superdup'r SD1002, Vivitro Systems Inc., Victoria, BC, Canada), which was modified to facilitate ultrasound interrogation by placing echo-transducer ports at the side of the LV apex and left atrium. The LV diaphragm, made of silicone rubber, had a hemi-ellipsoid shape (long-axis dimension = 53 mm, base diameter = 44 mm, thickness = 0.6 mm). The normal baseline LV volume contained 150 ml of saline. The hydraulic chamber surrounding the LV diaphragm was filled with distilled water. The LV diaphragm was contracted and expanded by a computer-programmed piston pump which controlled the volume of the hydraulic fluid. Pericardial bileaflet bio-prosthetic valves (diameter = 21 mm) were mounted at the aortic and mitral valve sites. Aortic, LV, and left atrial pressure, and aortic or mitral flow rate were monitored during experiments.

### Waveform programming

**Figure 1:**
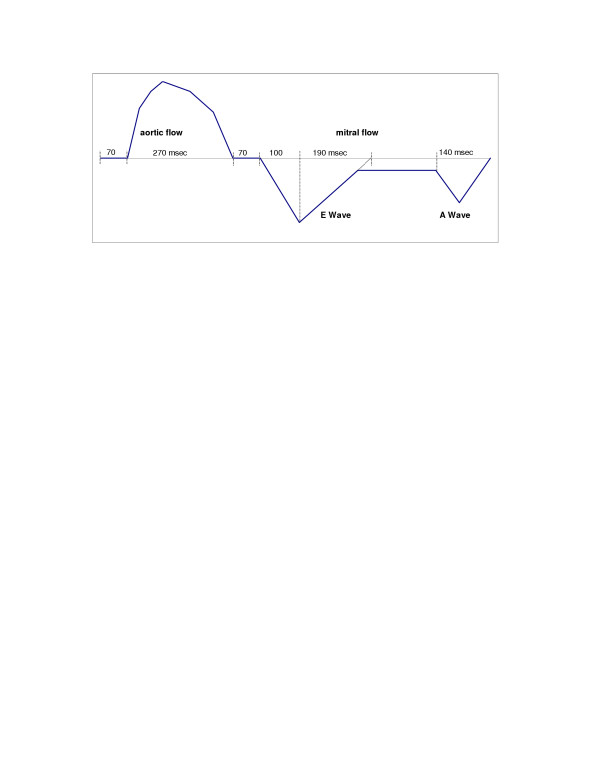
Type 1 transmitral flow velocity diagram: Constant diastasis flow. Heart rate = 60/min.

Aortic and mitral waveforms were programmed to mimic various physiological conditions. We programmed two different types of transmitral waveforms: Type 1 (with constant low velocity diastasis flow) and Type 2 (no mitral flow during the diastasis period; not shown in Figure 1) (Figure 1). Although HR was varied, transmitral waveforms were programmed to keep E (early diastolic) and A (late diastolic, or atrial) wave shape, acceleration time, and deceleration time constant at the mitral valve site. Aortic flow durations were set at 35% of cycle length at HR = 40 and 50/min, 27% at 60/min, 31% at 70/min, and 36% at 80/min.

### CMD measurements

CMD measurements were performed using a Vivid ultrasound unit (General Electric Vingmed Ultrasound, Horten, Norway). The transducer (2.5 MHz) was positioned at the LV apex site. The distance between transducer and mitral valve was 7.6 cm. The CMD red-blue interface (aliasing) velocity was set at 92 cm/sec to avoid signal "bleeding". CMD sweep speed was 200 mm/sec. The echocardiography machine settings were kept constant during all experiments. All CMD images were recorded on an optical disc or CD-ROM, and off-line analysis was performed using commercial software (EchoPac for Vivid 7). The Vp was measured as the slope of the first aliasing velocity from the mitral annulus to 4 cm distal in the left ventricle in early diastole. Vp measurements were performed 10 times for each flow condition.

### Hemodynamic variables

The following ranges of hemodynamic variables were studied: 1) stroke volume (SV): 50, 60, 70, 80, and 90 ml; 2) heart rate (HR): 40, 50, 60, 70, and 80/min; 3) baseline LV volume (LVV): 130, 150, 180, 200, and 200 ml; 4) LV compliance: five conditions; and 5) transmitral flow (TMF) waveforms (Type 1: featuring constant low diastasis flow between early and late diastolic waves and Type 2: featuring no diastasis flow). LVV was changed by adding distilled water to, or sucking hydraulic liquid from, the hydraulic chamber. Adding 0, 10, 20, 30, or 40 ml of air into the hydraulic chamber and sucking the same volume of hydraulic liquid from the hydraulic chamber changed LV compliance – specifically, the greater the volume of air, the greater the LV compliance. Baseline conditions were: Type 1 waveform, SV = 70 ml, HR = 60/min, LVV = 150 ml, and an LV compliance with 0 ml of air in the hydraulic chamber. LV compliance was calculated as volume change divided by pressure change during the period from the LV-left atrial pressure crossover-point to the minimum LV pressure in early diastole. The LV compliance (ml/mmHg) under 5 experimental conditions was calculated as follows: 0 ml air, 8.32 × 10^-3^; 10 ml air, 9.75 × 10^-3^; 20 ml air, 16.34 × 10^-3^; 30 ml air, 17.05 × 10^-3^; and 40 ml air, 19.92 × 10^-3^.

### Measurement Variability

Vp measurements were made using commercially available software. Vp was measured on two separate occasions on 26 images stored on disc by one reader to estimate intra-reader measurement variability. A second observer, blinded to the measurements made by the first reader, also measured Vp on these 26 images to calculate inter-reader variability.

### Statistical analysis

The association of hemodynamic parameters with Vp was analyzed using Student's paired *t *test and univariate regression analysis. All calculated *P *values were two-tailed, and a value of *P *< 0.05 was considered to indicate statistical significance.

## Results

### Measurement variability

The mean difference (+ or - SD) of Vp measurements for the same reader was 1.56 ± 0.88 cm/sec (2.0 ± 1.1% mean intra-reader variability). The mean difference (± SD) of Vp measurements between two readers was 1.79 ± 1.16 cm/sec (2.2 ± 1.4% mean inter-reader measurement variability).

### Relationship between hemodynamic variables and Vp

**Figure 2:**
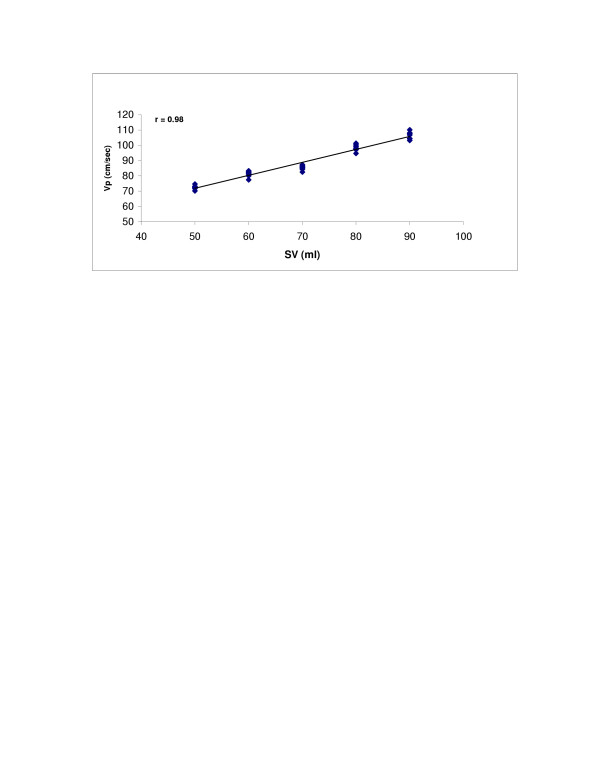
This graph shows a strong relationship between stroke volume (SV) and flow propagation velocity (Vp) (r = 0.98, p < 0.0001).

**Figure 3:**
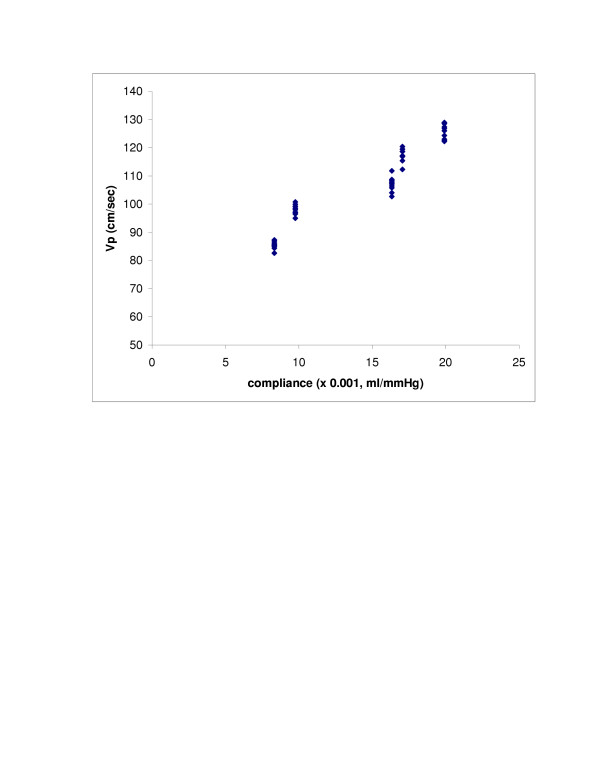
This graph shows an excellent correlation between LV compliance and Vp (r = 0.95, p < 0.0001).

**Figure 4:**
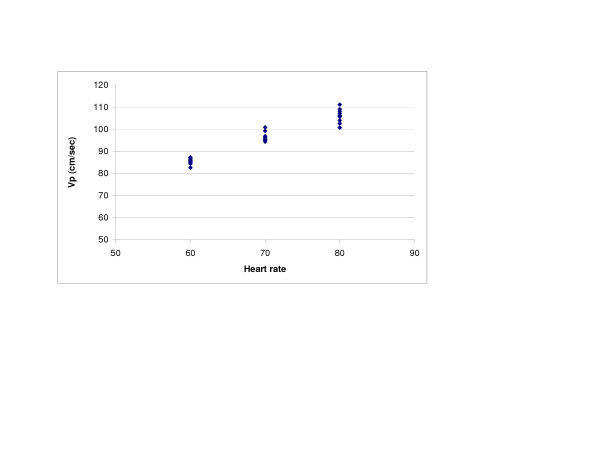
Relationship between heart rate (HR) and Vp with a Type 1 transmitral flow pattern: There was a good correlation between HR and Vp (r = 0.97, p < 0.0001).

**Figure 5:**
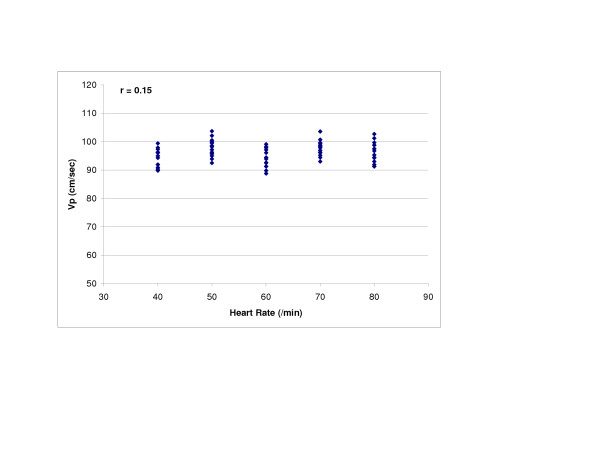
Relationship between heart rate (HR) and Vp with a Type 2 transmitral flow pattern: HR was not associated with Vp (r = 0.15, p = NS).

**Figure 6:**
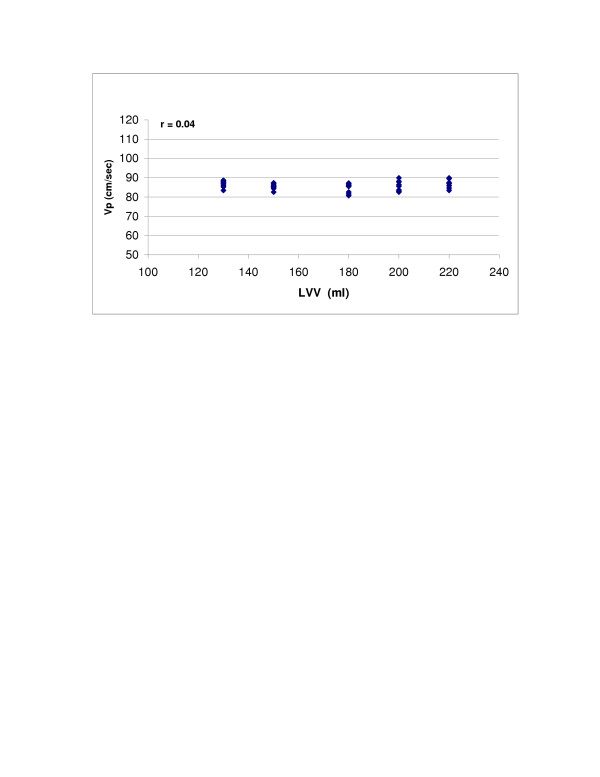
Relationship between LV volume (LVV) and Vp: Under low compliance conditions (no added air) and 70 ml stroke volume, LVV was not associated with Vp (r = 0.04, p = NS).

**Figure 7:**
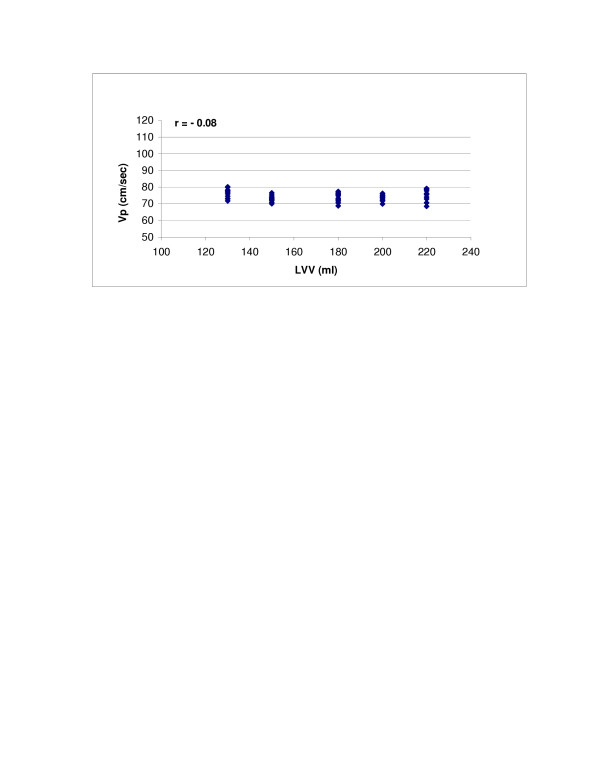
Relationship between LVV and Vp: Under low compliance conditions and 50 ml stroke volume, LVV was not associated with Vp (r = -0.08, p = NS).

**Figure 8:**
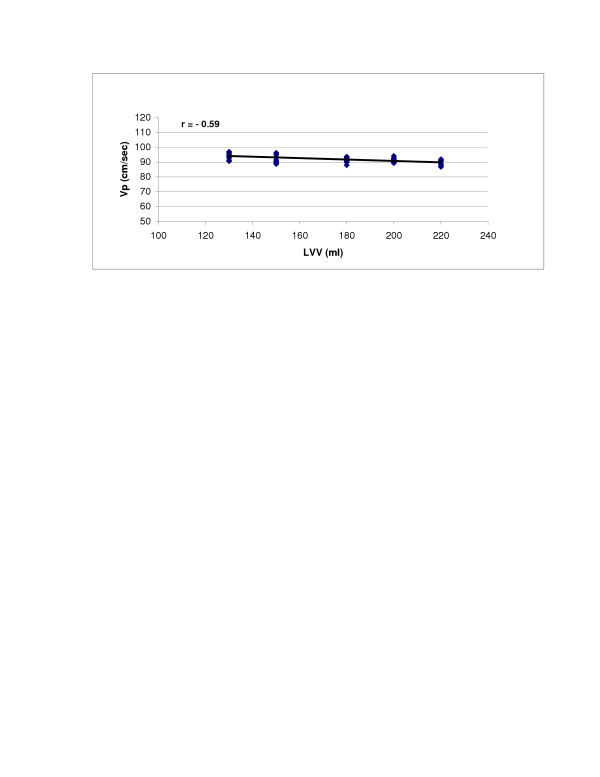
Relationship between LVV and Vp: Under high compliance conditions (40 ml air in hydraulic chamber) and SV = 50 ml, LVV was inversely associated with Vp (r = -0.59, p < 0.0001).

**Figure 9:**
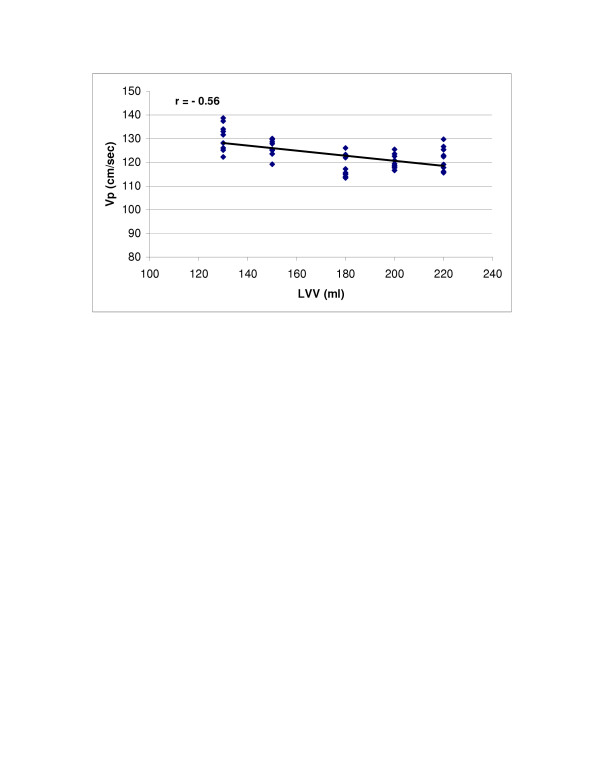
Relationship between LVV and Vp: Under high compliance conditions and SV = 70 ml, LVV was inversely associated with Vp (r = -0.56, p < 0.0001).

SV (r = 0.98, p < 0.0001) and LV compliance (r = 0.95, p < 0.0001) both showed excellent correlations with Vp (Figures 2 and 3). With Type 1 transmitral waveform conditions, Vp increased as HR increased (r = 0.97, p < 0.0001) (Figure 4). However, with Type 2 waveform conditions, HR was not associated with Vp (r = 0.15, p = NS) (Figure 5). The LV volume was not associated with Vp at SV 50 ml (r = -0.08, p = NS) and SV 70 ml (r = 0.04, p = NS) under low compliance conditions (no added air, Figures 6 and 7). However, under high compliance conditions (40 ml air in the hydraulic chamber), LV volume was inversely associated with Vp at SV 50 ml (r = 0.59, p < 0.0001) and SV 70 ml (r = -0.56, p < 0.0001, Figures 8 and 9).

## Discussion

### Stroke Volume and Vp

Many investigators have studied diastolic suction. They have characterized diastolic suction as ventricular filling under zero source pressure for filling or peak diastolic negative pressure. The elastic recoil of the relaxing ventricular wall is considered to be the source of the ventricular suction force [5,6]. Courtois reported that the intraventricular pressure gradient (IVPG) between the apex and base of the left ventricle during early diastolic filling was reduced in myocardial ischemia cases; they speculated that IVPGs were related to elastic recoil [7]. Greenberg reported that IVPG was correlated with flow propagation velocity [8]. Smiseth demonstrated that IVPG also correlated with peak early transmitral flow and stroke volume in canine studies [9]. More recently, in their clinical investigation, Ohte showed a good relationship between Vp and LV end-systolic volume or LV ejection fraction [10]. The positive relationship between Vp and stroke volume documented in the current study is consistent with these reports.

### LV compliance and Vp

Using computer simulations, Lemmon showed that the effect of delayed relaxation was to decrease the early filling propagation, and this decrease was larger when the stiffness of the ventricle was increased [11]. Vierendeels also demonstrated that higher LV stiffness was associated with a smaller Vp in their computer simulations [12,13]. Our findings are consistent with these previous studies.

### Heart rate and early diastolic LV filling

The difference in HR relationship to Vp with our two transmitral waveform models (Type 1 and Type 2) may be due to the fact that the waveform program software automatically modifies transmitral velocity-time integral to be equal to the aortic velocity-time integral. There is no in vivo study confirming a HR effect on Vp. However, some previous clinical studies and human or canine studies employing atrial pacing demonstrated that increasing HR was not associated with a change in mitral peak E velocity on pulsed wave Doppler [14-18]. Cheng reported that in their canine studies using atrial pacing, as HR increased from 100 to 160/min, left atrial pressure dropped in a monotonic fashion and τ also decreased [19]. Therefore, relaxation filling was unchanged at HR up to 160/min, consistent with the opposite and counterbalancing influences of HR and τ [19]. In our studies, left atrial pressure was constant though HR changed. However, in an epidemiologic study [20] and in an atrial pacing study of patients with DDD pacemakers [21], the transmitral peak E velocity decreased as HR increased. Many factors affect the transmitral flow profile, and this discrepancy may be based on other factors (e.g., a normal vs. pathological heart, myocardial stiffness, preload, and LV geometry).

### LV volume and Vp

Steen reported, in a mechanical LV model similar to ours, that a larger ventricle had a higher Vp than did a smaller ventricle for the same transmitral peak E wave velocity [22]. The difference between Steen's results and ours may be due to: 1) the presence of an LV outflow tract in our LV model, and 2) the fact that our model included a flow circuit, but their simple model did not include serial LV inflow, which would have mitigated any adverse effect of inertia on the LV inflow profile. In their finite element model, Sunagawa reported that the combination of increased LV mass, low stiffness and low strain axis parameter, was associated with increased SV with ventricular volume reduction [23]. Our results related to the LVV and Vp, are consistent with the latter study.

## Study limitations

Our hydraulic LV model had various limitations in simulating the human left ventricle. Specifically, the LV had negligible mass, no papillary muscles and, higher volume compared with the normal human left ventricle, and lacked the LV twisting motion and left atrial contraction with changes in left atrial volume. Also, during LV filling, the position and size of the mitral valve annulus were fixed in our model.

## Conclusion

We used a mechanical LV model to study how Vp measured by color M-mode Doppler echocardiography varied with changes in stroke volume, heart rate, LV volume, LV compliance, and mitral waveform. Our results documented important relationships between various physiologic parameters and Vp. These studies of Vp should help to elucidate the physiology of early diastolic LV filling. Further studies are needed to investigate the relationship between factors such as left atrial pressure, severity of mitral regurgitation, and mitral annulus size and Vp.

## Declaration of Competing Interests

Drs. Toshihiro Ogawa, Ajit Yoganathan, Cheryl Nordstrom, Julius Gardin, and Renee Bess all declare that they have no competing interests. Dr. David Walker and Mr. Lawrence Scotten state that during the time of the performance of these studies, they were employees of Vivitro Systems, Inc., which was the manufacturer of the in vitro flow model (Superdup'r SD1002) used in this study.
